# Supplementary material for: Methylation of ESCRT-III components regulates the timing of cytokinetic abscission
Source: Nat Commun. 2024 May 13;15:4023. doi: 10.1038/s41467-024-47717-3 (PMC11091153; doi:10.1038/s41467-024-47717-3)
Supplement: Supplementary file 1 — Supplementary Information [file 41467_2024_47717_MOESM1_ESM.pdf]

Title

Methylation of ESCRT-III components regulates the timing of cytokinetic abscission

Authors

Aurélie Richard[1*], Jérémy Berthelet[1*], Delphine Judith[2], Tamara Advedissian[3], Javier Espadas[4], Guillaume Jannot[1], Angélique Amo[1], Damarys Loew[5], Berangere Lombard[5], Alexandre G. Casanova[6], Nicolas Reynoird [6], Aurélien Roux [4], Clarisse Berlioz-Torrent [2], Arnaud Echard [3], Jonathan B. Weitzman[1], Souhila Medjkane[1]

Affiliations

[1] Université Paris Cité, CNRS, UMR7126 Epigenetics and Cell Fate, F-75013 Paris, France

[2] Université Paris Cité, Inserm, CNRS, Institut Cochin, F-75014 Paris, France

[3] Institut Pasteur, Université Paris Cité, CNRS UMR3691, Membrane Traffic and Cell Division Unit, 25-28 rue du Dr Roux, F-75015 Paris, France

[4]Department of Biochemistry, University of Geneva, Geneva CH-1211, Switzerland.

[5] Institut Curie, PSL Research University, Centre de Recherche, CurieCoreTech Mass Spectrometry Proteomics, F-75005 Paris, France

[6] Université Grenoble Alpes, CNRS UMR5309, INSERM U1209, Institute for Advanced Biosciences, 38000 Grenoble, France

Address correspondence to:

UMR 7216 Epigenetics and Cell Fate

Université Paris Cité, CNRS

Bâtiment Lamarck, Case 7042, 35 rue Hélène Brion

75205 PARIS cedex 13, France

email: souhila.medjkane@u-paris.fr

https://orcid.org/0000-0001-7973-7707

* These authors contributed equally to this work

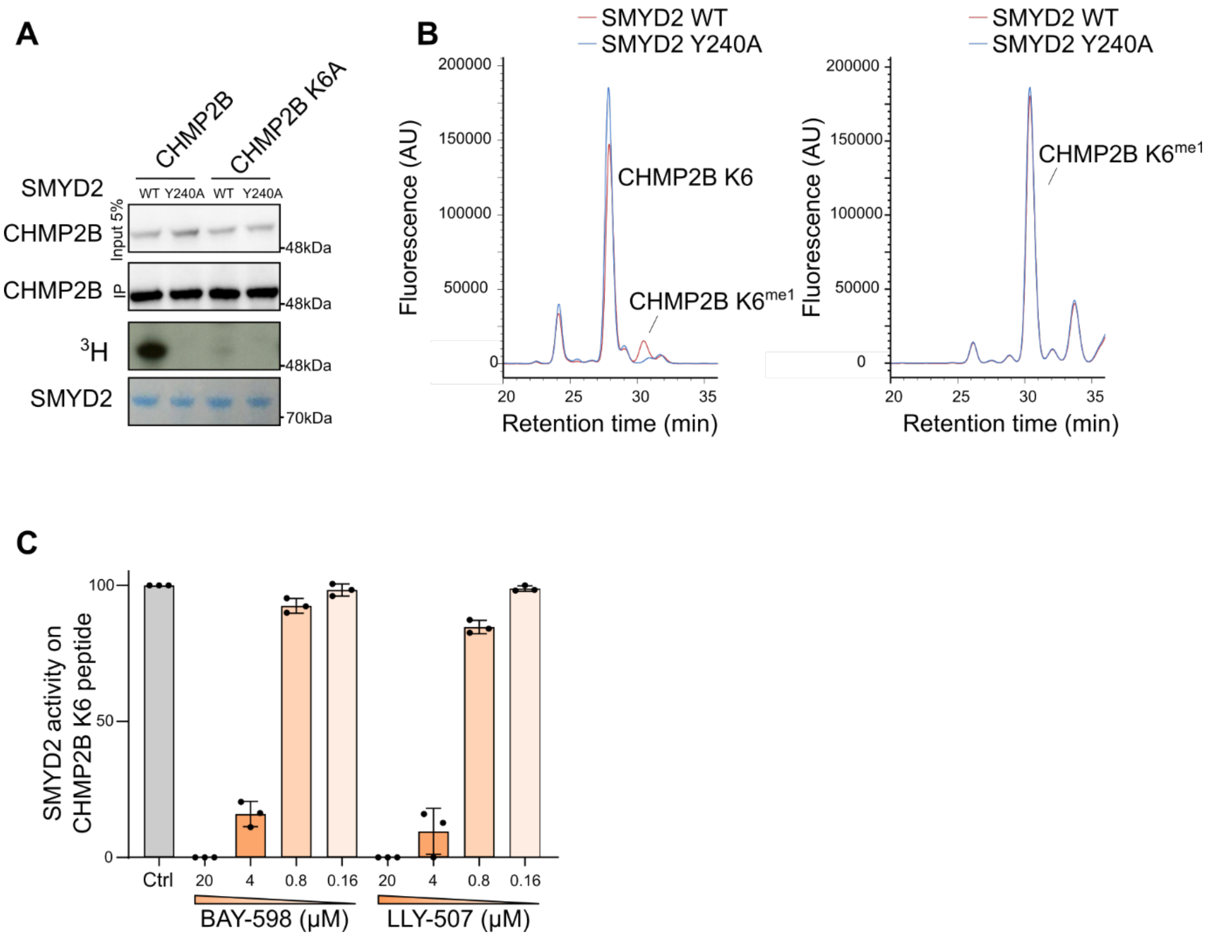

Supplementary Fig. 1. SMYD2 monomethylates CHMP2B K6

(**A**) CHMP2B_GFP, CHMP2B K6A_GFP were transiently transfected in HeLa cells and immunoprecipitated with GFP antibody. The immunoprecipitated proteins were incubated with recombinant active SMYD2 or SMYD2 Y240A dead catalytic mutant. Samples were then subjected to SDS-PAGE, and the total proteins were visualized using Coomassie staining. Gel was later dried and methylated proteins were detected by autoradiography. (**B**) Recombinant SMYD2 (WT or catalytic dead Y240A mutant) were incubated in the presence of CHMP2B K6 FAM-ASLFKKKTVD-NH2 (left) or CHMP2B $K6_{me1}$ FAM-ASLFK_{me1}KKTVD-NH2 (right). Resulting peptides were detected by reverse phase-ultra-fast liquid chromatography (RP-UFLC) and monitored by fluorescence emission at 530 nm. (**C**) Recombinant SMYD2 was incubated in the presence of CHMP2B substrate peptide FAM-ASLFKKKTVD-NH2 and different concentrations of BAY-598 or LLY-507, two SMYD2 inhibitors. SMYD2 residual activity was measured by RP-UFLC and quantified according to material and methods. Barplots and error bars represent the means and the SD of 3 independent replicates.

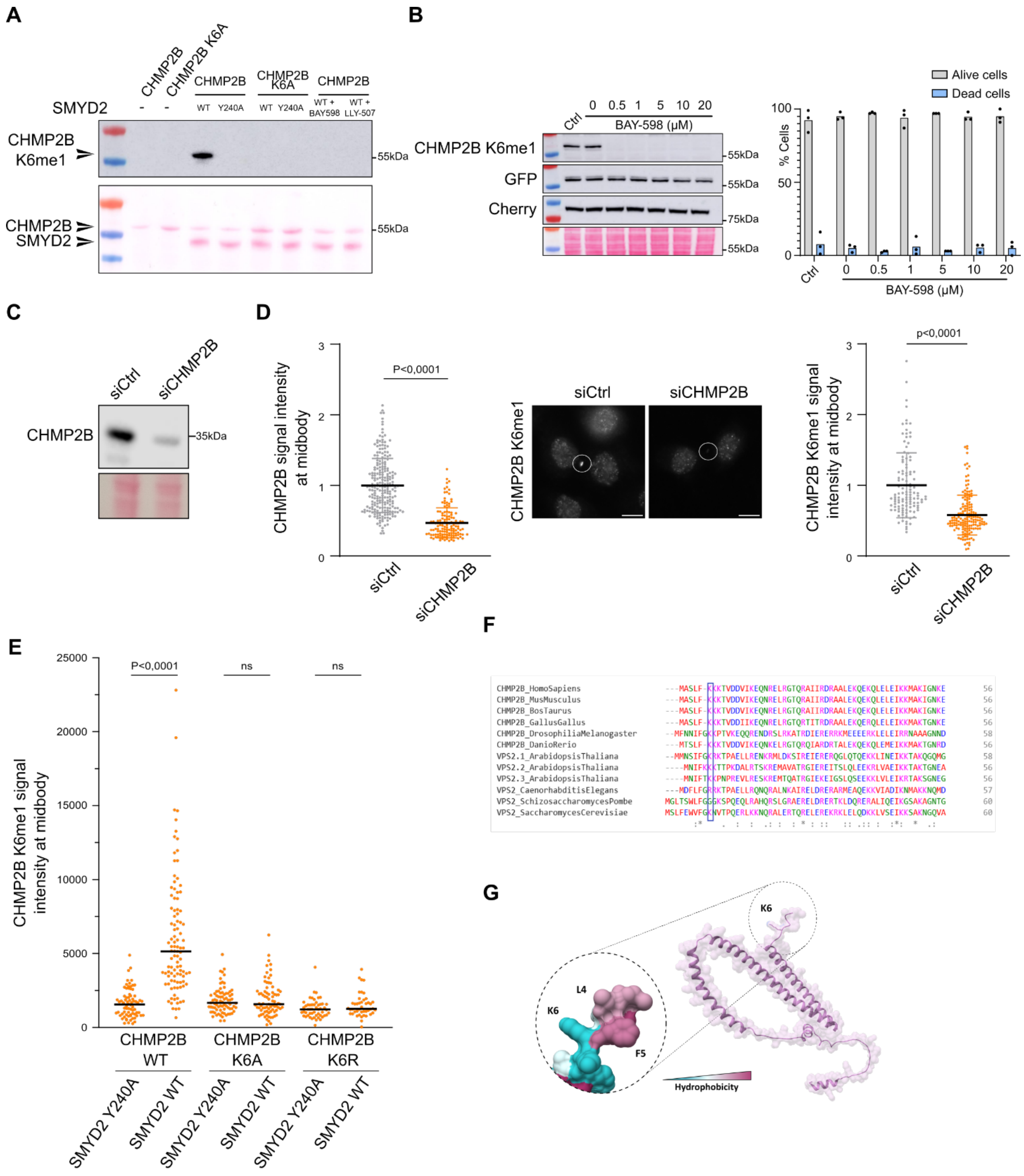

Supplementary Fig.2 . CHMP2B K6 is a conserved residue and its methylation is revealed by the anti-CHMP2B K6me1 antibody

(**A**) Recombinant SMYD2 (WT or catalytic dead Y240A mutant) were incubated with recombinant CHMP2B (WT or K6A mutant) and with 1 µM of BAY-598 or LLY-507. The samples were then separated by SDS-PAGE, transferred onto a nitrocellulose membrane, and the total proteins were visualized by Ponceau staining. The CHMP2B K6 mono-methylation was then detected using CHMP2B K6me1 antibody. Representative image of N=3 experiments. (**B**) (left) Protein extracts from HeLa cells lines stably expressing CHMP2B_GFP and Cherry_SMYD2 and treated for 48 hrs with DMSO or SMYD2 inhibitor (BAY-598 10µM) were blotted for CHMP2B K6me1 or GFP antibodies. Loading control: Ponceau red. (right) Cell viability was assessed. N=3 (**C**) Western

blots of protein extracts from HeLa cell lines stably expressing SMYD2_GFP treated with control or CHMP2B siRNAs revealed with CHMP2B. Loading control: Ponceau red staining. (**D**) Quantification of CHMP2B signal (left) or CHMP2B K6me1 signal (right) at the midbody in HeLa cells stably expressing SMYD2_GFP treated with control or CHMP2B siRNAs (N=4 n>117 midbodies (CHMP2B K6me1) or N=3 n>134 midbodies (CHMP2B), mean ± SD, Two-sided Mann–Whitney test). (Middle) Representative cell images of CHMP2B K6me1 immunostaining of HeLa cells stably expressing SMYD2_GFP treated with control or CHMP2B siRNAs. The midbody region is indicated by a white circle. Scale bar =5 µm. (**E**) Quantification of CHMP2B K6me1 signal at the midbody in HeLa cells stably expressing CHMP2B_WT-GFP, CHMP2B K6A-GFP or CHMP2B K6R-GFP and transfected either with SMYD2 WT or SMYD2 Y240A mutant and CHMP2B siRNA (Two-sided multiple unpaired t tests, N=3, n=44-101 midbodies). (**F**) Multiple alignments of the protein sequences of CHMP2B N-terminal tails from various species including *Homo sapiens, Mus musculus, Bos taurus, Gallus gallus, Drosophilia melanogaster, Danio rerio, Arabidopsis thaliania, Caenorhabditis elegans, Schizosaccharomyces pombe* and *Saccharomyces cerevisiae* using Clustal Omega. Residues are colored according to their physicochemical properties. Blue rectangle shows the K6 residue. (**G**) Global representation of the structure of the human CHMP2B generated using Alphafold2 and visualized using Chimera. The position of the lysine 6 residue of interest is displayed. The zoomed panel shows a hydrophobicity surface representation of the N-terminal structure of CHMP2B based on the Eisenberg hydrophobicity scale. The red surface indicates the highest hydrophobicity, while the blue surface represents the lowest hydrophobicity.

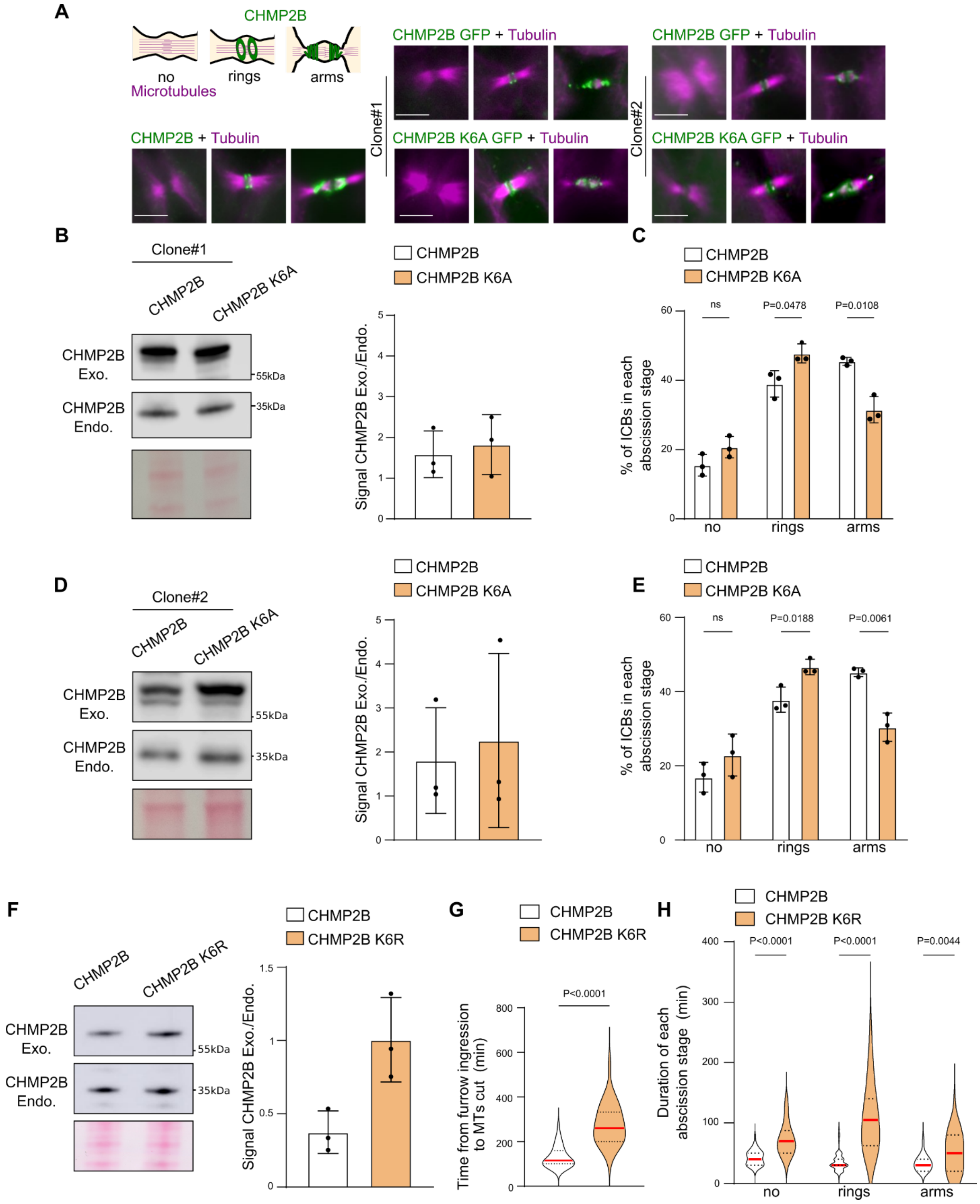

Supplementary Fig. 3. CHMP2B K6 is required for the progression of CHMP2B to the abscission site

(**A**) Top left: Schematic representation of CHMP2B localization during cytokinesis made using Inkscape software. In the early abscission stage, CHMP2B is absent from the intercellular bridge. Subsequently, CHMP2B is recruited as two rings at both sides of the midbody. Finally, CHMP2B extends along the midbody arms during the late abscission stage until the membrane is severed.

These three stages were employed to evaluate the progression of cytokinesis. Bottom left: representative cell images of endogenous CHMP2B (green) and alpha-tubulin (magenta) immunostaining in HeLa cells in each abscission stage. Right panels: representative cell images showing alpha-tubulin staining (magenta) in cell lines stably expressing CHMP2B_GFP or CHMP2B K6A_GFP from two different clone combinations (clone #1 and clone #2) at each abscission stage. Scale bar = 5 μm. (**B**) Left panel: Western blots of protein extracts from HeLa cell lines stably expressing CHMP2B_GFP or CHMP2B K6A_GFP (clone #1) probed with CHMP2B antibody. Right panel: Quantification of exogenous (Exo.) on endogenous (Endo.) CHMP2B signal for CHMP2B_GFP or CHMP2B K6A_GFP cell lines. Loading control: Ponceau red staining. (**C**) Quantification of ICBs in each abscission stage defined according to CHMP2B_GFP or CHMP2B K6A_GFP localization in HeLa cell lines stably expressing CHMP2B_GFP or CHMP2B K6A_GFP (clone #1) (N=3 n>100 ICBs counted/N, mean ± SEM, Two-sided multiple unpaired t test, ns = non-significant (p>0.05)). (**D**) Left panel: Western blots of protein extracts from HeLa cell lines stably expressing CHMP2B_GFP or CHMP2B K6A_GFP (clone #2) probed with CHMP2B antibody. Right panel: Quantification of exogenous (Exo.) on endogenous (Endo.) CHMP2B signal for CHMP2B_GFP or CHMP2B K6A_GFP cell lines. Loading control: Ponceau red staining. (**E**) Quantification of ICBs in each abscission stage defined according to CHMP2B_GFP or CHMP2B K6A_GFP localization in HeLa cell lines stably expressing CHMP2B_GFP or CHMP2B K6A_GFP (clone #2) (N=3 n>100 ICBs counted/N, mean ± SEM, Two-sided multiple unpaired t test, ns = non-significant (p>0.05)). (**F**) Western blots of protein extracts from HeLa cell lines stably expressing CHMP2B_GFP or CHMP2B K6R_GFP probed with CHMP2B antibody. Loading control: Ponceau red staining. (**G**) Quantification of the abscission time of HeLa cell lines stably expressing CHMP2B_GFP or CHMP2B K6R_GFP (calculated from furrow ingression formation to the microtubule cut), (N=3, n>50, median (red lines) and quartiles (dotty lines), Two-sided Mann–Whitney test). (**H**) Duration of each abscission stage measured from live cell imaging performed in CHMP2B_GFP or CHMP2B K6R_GFP expressing cells (N=3, n>50, median (red lines) and quartiles (dotty lines), Two-sided Mann–Whitney test).

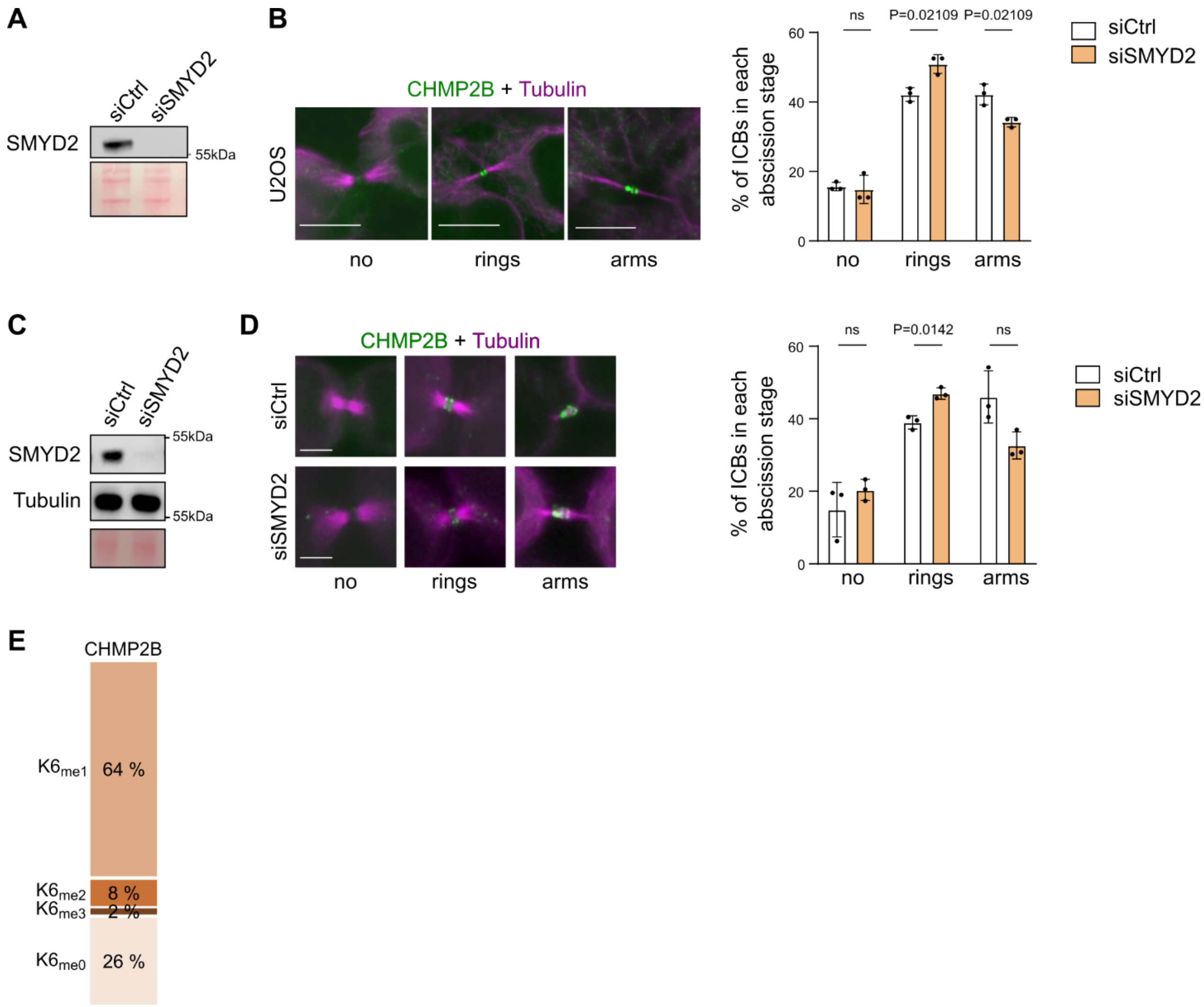

Supplementary Fig. 4. SMYD2 regulates the localization of CHMP2B at the ICB
(**A**) Western blots of protein extracts from HeLa cells treated with control or SMYD2 siRNAs revealed with SMYD2 or tubulin antibodies. Loading controls: Tubulin and Ponceau red staining. (**B**) Left panels: representative cell images of endogenous CHMP2B (green) and alpha-tubulin (magenta) immunostaining in HeLa cells treated with control or SMYD2 siRNAs representing different abscission stages according to CHMP2B localization: no CHMP2B, CHMP2B as rings at the midbody, and CHMP2B both at the midbody and at the abscission site. Scale bar = 5 µm. Right panel: quantification of ICBs in each abscission stage defined according endogenous CHMP2B localization in HeLa cells treated with either control or SMYD2 siRNAs (N=3 n>50 ICBs counted/N, median (red lines) and quartiles (dotty lines), Two-sided multiple unpaired t test, ns = non-significant (p>0.05)). (**C**) Western blots of protein extracts from U2OS cells treated with control or SMYD2 siRNAs revealed with CHMP2B antibody. Loading control: Ponceau red staining. (**D**) Left panels: representative cell images of endogenous CHMP2B (green) and alpha-tubulin (magenta) immunostaining in U2OS cells treated with control siRNA showing the three abscission stages according to CHMP2B localization. Scale bar = 10 µm. Right panel: quantification of ICBs in each abscission stage defined according to endogenous CHMP2B localization in U2OS cells treated with either control or SMYD2 siRNAs (N=3, n>40 ICBs counted/N, mean ± SEM, Two-sided multiple unpaired t test, ns = non-significant (p>0.05)). (**E**) The methylation levels of recombinant CHMP2B K6 incubated with SMYD2 protein was measured by mass spectrometry as indicated.

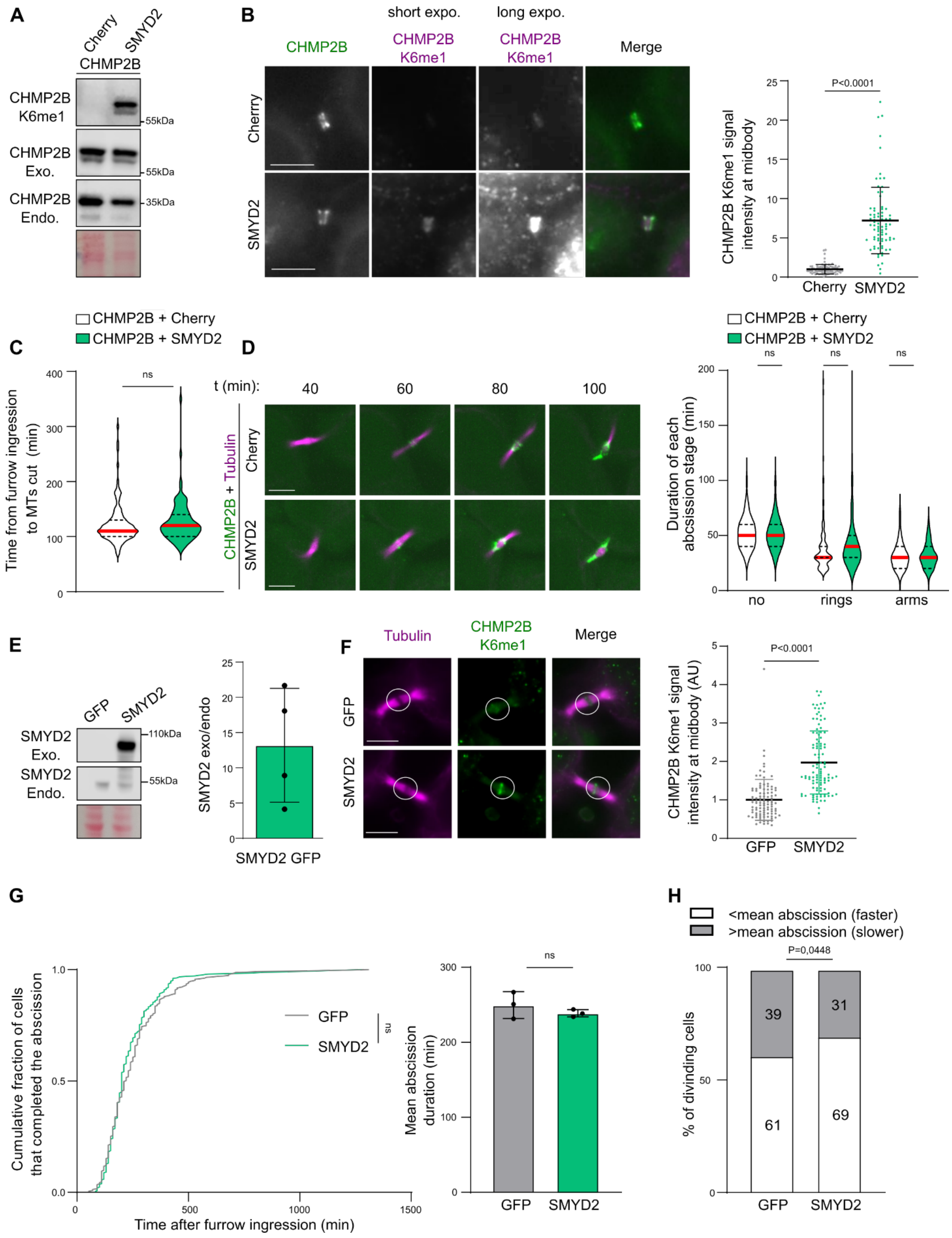

Supplementary Fig. 5. SMYD2 ectopic expression increases CHMP2B K6 methylation

(**A**) Western blots of protein extracts from HeLa cell lines stably expressing CHMP2B_GFP with Cherry or Cherry_SMYD2 revealed with CHMP2B K6me1 or CHMP2B antibodies. Loading control: Ponceau red staining. (**B**) Left panels: representative cell images. CHMP2B K6me1 signal is shown with longer exposure time. Scale bar = 5 µm. Right panel: quantification of the CHMP2B

K6me1 signal at the midbody of the indicated HeLa cell lines (N=3, n>80 midbodies/condition, mean ± SD, Two-sided Mann-Whitney test, ns = non-significant (p>0.05)). (**C**) Quantification of the abscission time of HeLa cells stably expressing CHMP2B_GFP with Cherry or with Cherry_SMYD2 (calculated from furrow ingression formation to the microtubule cut). (N=3, n>87 cells, median (red lines), and quartiles (dotted lines), Two-sided Mann–Whitney test). (**D**) Left panels: live cell imaging of the indicated HeLa cell lines. Time 00 is set at time point prior the ICB formation. Last time point corresponds to the microtubule cut, considered here as the abscission. Scale bar = 5 µm. Right panel: Duration of each abscission stage measured from live cell imaging (N=3, n>87 cells, median (red lines), and quartiles (dotted lines), Two-sided Mann–Whitney test). (**E**) Left panels: Western blots of protein extracts from the indicated HeLa cell lines revealed with SMYD2 antibody. Loading control: Ponceau red. Right panel: quantification of the ratio between exogenous SMYD2_GFP and endogenous SMYD2 signal, N=4 independent western blots). (**F**) Left panels: Representative cell images of CHMP2B K6me1 (green) and alpha-tubulin (magenta) immunostaining. The white circle shows the midbody. Scale bar = 5 µm. Right panel: quantification of the CHMP2B K6me1 signal at the midbody of the indicated HeLa cell lines (N=3 n>101 midbodies/condition, mean ± SD, Two-sided Mann-Whitney test). (**G**) Left panels: distribution of the abscission time measured by phase-contrast time-lapse microscopy (N=3, n>231 cells, Two-sided Kolmogorov–Smirnov test, ns = non-significant (p>0.05)) and mean abscission duration (N=3, mean ± SD, t test). (**H**) Quantification of the % of dividing cells completing their abscission faster (<mean abscission) or longer (>mean abscission) than the mean abscission time of GFP control cells (Pool of N=3, n>231 cells, Two-sided Chi2 test).

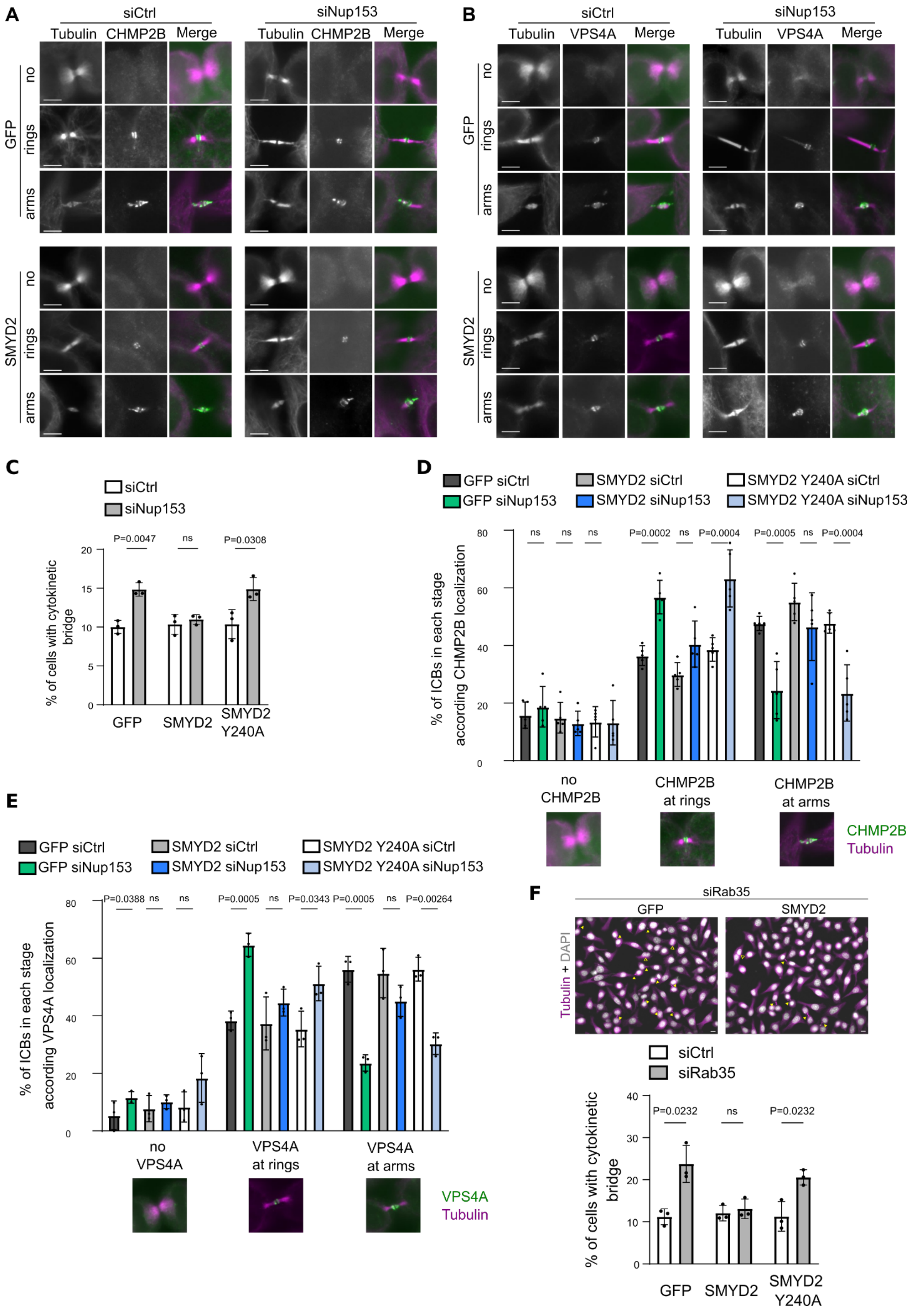
A
siCtrl
siNup153
Tubulin
CHMP2B
Merge
GFP
SMYD2
no
rings
arms
B
VPS4A
C
siCtrl
siNup153
% of cells with cytokinetic bridge
P=0.0047
ns
P=0.0308
GFP
SMYD2
SMYD2 Y240A
D
GFP siCtrl
GFP siNup153
SMYD2 siCtrl
SMYD2 siNup153
SMYD2 Y240A siCtrl
SMYD2 Y240A siNup153
% of ICBs in each stage according CHMP2B localization
P=0.0002
P=0.0004
P=0.0005
P=0.0004
no CHMP2B
CHMP2B at rings
CHMP2B at arms
CHMP2B
Tubulin
E
% of ICBs in each stage according VPS4A localization
P=0.0388
P=0.0005
P=0.0343
P=0.0005
P=0.00264
no VPS4A
VPS4A at rings
VPS4A at arms
VPS4A
Tubulin
F
siRab35
Tubulin + DAPI
siCtrl
siRab35
P=0.0232
ns
P=0.0232

Supplementary Fig. 6. SMYD2 accelerates abscission timing when cytokinesis is challenged

(**A**) Representative cell images of endogenous CHMP2B (green) and alpha-tubulin (magenta) immunostaining in HeLa cell lines stably expressing GFP or SMYD2_GFP treated with control or Nup153 siRNAs, showing the three abscission stages according to CHMP2B localization. Scale bar= 5 µm. (**B**) Representative cell images of endogenous VPS4A (green) and alpha-tubulin (magenta) immunostaining in HeLa cell lines stably expressing GFP or SMYD2_GFP treated with control or Nup153 siRNAs, showing the three abscission stages according VPS4A localization. Scale bar= 5 µm. (**C**) Quantification of the percentage of cytokinetic cells in HeLa cell lines stably expressing GFP, SMYD2_GFP or SMYD2 Y240A_GFP (dead catalytic mutant) treated with control or Nup153 siRNAs. (N=3, n>300 cells counted/N, mean ± SD, Two-sided multiple unpaired t test). (**D**) Quantification of ICBs with either no CHMP2B, CHMP2B as rings at the midbody, and CHMP2B both at the midbody and at the abscission site in HeLa cell lines stably expressing GFP, SMYD2_GFP or SMYD2 Y240A_GFP treated with control or Nup153 siRNAs (N=5, n>40 ICBs counted/N, mean ± SD, Two-sided multiple unpaired t test, ns = non-significant (p>0.05)). (**E**) Quantification of ICBs with either no VPS4A, VPS4A at midbody rings, and VPS4A both at the midbody and at the abscission site in HeLa cell lines stably expressing GFP, SMYD2_GFP or SMYD2 Y240A_GFP treated with control or Nup153 siRNAs (N=3, n>50 ICBs counted/N, Two-sided multiple unpaired t test). (**F**) Top panels: representative cell images of HeLa cell lines stably expressing GFP (left) or SMYD2_GFP (right) treated with Rab35 siRNA stained for alpha-tubulin. Empty arrows show cytokinetic cells connected by an ICB, full arrows show binucleated cells. Scale bar = 10 µm. Bottom panels: Quantification of the percentage of cytokinetic cells in HeLa cell line stably expressing GFP, SMYD2_GFP or SMYD2 Y240A_GFP treated with control or Rab35 siRNAs. (N=3, n>300 cells counted/N, mean ± SD, Two-sided multiple unpaired t test).

Plasmid Name	Insert	Origin	Details
psPAX2	Gag+pol	Addgene	
pMD2.G	VSV-G Env	Addgene	
pENG1	GFP	Ref[65]	
pENG1 SMYD2 GFP	human SMYD2	this paper	GFP in C ter
pENG1 SMYD2 Y240A GFP	human SMYD2 Y240A	this paper	GFP in C ter
pENG1 Cherry	Cherry	this paper	
pENG1 Cherry SMYD2	human SMYD2	this paper	Cherry in N ter
pENG1 CHMP2B_GFP	human CHMP2B siRNA res	this paper	GFP in C ter
pENG1 CHMP2B K6A GFP	human CHMP2B K6A siRNA res	this paper	GFP in C ter
pENG1 CHMP2B K6R GFP	human CHMP2B K6R siRNA res	this paper	GFP in C ter

Supplementary Table 1
Lentivectors used in this study

Plasmid Name	Insert	Species	Origine	Details
pDonor207	CHMP2B	human	this paper	
pDonor207	SMYD2	human	this paper	
pDonor207	SMYD2 Y240A	human	this paper	
pDest-eGFP-C1	CHMP2B	human	this paper	GFP in N ter
pDest-eGFP-C1	SMYD2	human	this paper	GFP in N ter
pDest-eGFP-C1	SMYD2 Y240A	human	this paper	GFP in N ter
pDest-mCherry-C1	SMYD2	human	this paper	Cherry in N ter
pCiNeo-3xFLAG	SMYD2	human	this paper	flag in N ter
pmRFP_LAP2_β_IRES_puro2b	LAP2 β	human	Daniel Gerlich lab ref [58]	RFP in N ter
pSUMO (LifeSensor)	SMYD2	human	this paper	6xHis in N ter
pGEX-6P-1 (Addgene)	CHMP2B	human	this paper	GST in N ter
pet28 MBP TEV(Addgene)	CHMP3, CHMP2B	human	this paper	6xHis in N ter

Supplementary Table S2
Plasmids used in this study

Name	Sense	Anti-Sense	Ref
SiLuciferase (ctrl)	CGUACGCGGAAUACUUCGA[dT][dT]	UCGAAGUAUUCCGCGUACG[dT][dT]	
siControl	D-001810-01 from Dharmacon		
siCHMP2B	UCGAGCAGCUUUAGAGAAA[dT][dT]	UUUCUCUAAAGCUGCUCGA[dT][dT]	Ref [35]
siSMYD2	GACAAUGAUAGCCUCGUAGUA[dT][dT]	UACUACGAGGCUAUCAUUGUC[dT][dT]	
siNup153	GGACUUGUUAGAUCUAGUU[dT][dT]	AACUAGAUCUAACAAGUCC[dT][dT]	Ref [52]
siRab35	UUUACUGUUCUUCGUGAGC[dT][dT]	GCUCACGAAGAACAGUAAA[dT][dT]	Ref [40]

Supplementary Table S3
siRNAs used in this study

Name	Seq	Mutated plasmid	Method
SMYD2 Y240A For	5’ [P]-TACCCAACGGAAGATAGAAATGACCGG 3’	SMYD2 in pDonor207	Phusion Site-Directed Mutagenesis Kit
SMYD2 Y240A Rev	5’ [P]-CAGGAGATCAATGGCGCTGGTAAAAAC 3’	SMYD2 in pDonor207	Phusion Site-Directed Mutagenesis Kit
SMYD2 Y240A For	5’GAGAGGAGGTTTTTACCAGCGCTATTGATCTCCTGTACCCAA 3’	SMYD2 in pSUMO	Agilent QuikChange XL site-directed mutagenesis kit
SMYD2 Y240A Rev	5’TTGGGTACAGGAGATCAATAGCGCTGGTAAAAACCTCCTCTC 3’	SMYD2 in pSUMO	QuikChange XL site-directed mutagenesis kit
CHMP2B K6A For	5'CTCATGGCGTCCCTCTTCGCGAAGAAAACCGTGGATGA 3'	CHMP2B in pGEX-6P-1	QuikChange XL site-directed mutagenesis kit
CHMP2B K6A Rev	5'TCATCCACGGTTTTCTTCGCGAAGAGGGACGCCATGAG 3'	CHMP2B in pGEX-6P-1	QuikChange XL site-directed mutagenesis kit
CHMP2B K6A For	5’ [P]-ATGTAATAAAGGAACAGAATCGAG 3’	CHMP2B in pDonor207	Phusion Site-Directed Mutagenesis Kit
CHMP2B K6A Rev	5’ [P]-CATCCACGGTTTTCTTCGCGAAGAGGGACGC 3’	CHMP2B in pDonor207	Phusion Site-Directed Mutagenesis Kit
CHMP2B siR For	5’ [P]-CTGCACTAGAAAAACAAGAAAAACAGCTGG 3'	CHMP2B or K6A in pDonor207	Phusion Site-Directed

			Mutagenesis Kit
CHMP2B siR Rev	5’ [P]-CACGGTCTCTGATTATAGCCCTCTGTGTAC 3'	CHMP2B or K6A in pDonor207	Phusion Site-Directed Mutagenesis Kit
CHMP2B K6R For	5'-GATGGCGTCCCTCTTCAGGAAGAAAACCGTGGATG-3'	CHMP2B in pDonor207	Phusion Site-Directed Mutagenesis Kit
CHMP2B K6R Rev	5'-CATCCACGGTTTTCTTCCTGAAGAGGGACGCCATC-3'	CHMP2B in pDonor207	Phusion Site-Directed Mutagenesis Kit
Y240A SMYD2 F	5’ [P] GAGAGGAGGTTTTTACCAGCGCTATTGATCTCCTGTACCCAA 3'	SMYD2 in pSUMO	QuikChange XL site-directed mutagenesis kit
Y240A SMYD2 R	5’ [P] TTGGGTACAGGAGATCAATAGCGCTGGTAAAAACCTCCTCTC 3'	CHMP2B in pSUMO	QuikChange XL site-directed mutagenesis kit
K6A CHMP2B F	5’ [P] CTCATGGCGTCCCTCTTCGCGAAGAAAACCGTGGATGA 3'	CHMP2B in pGEX-6P-1	QuikChange XL site-directed mutagenesis kit
K6A CHMP2B R	5’[P] TCATCCACGGTTTTCTTCGCGAAGAGGGACGCCATGAG 3'	CHMP2B in pGEX-6P-1	QuikChange XL site-directed mutagenesis kit

Supplementary Table S4

Primers used in this study to perform mutagenesis

	Name	Ref	Dilution
**Antibodies used for WB**	gfp	ab290 abcam	1/5000
	flag	sigma A 8592	1/5000
	CHMP2B	AB 226298	1/500
	CHMP2B K6me1	Home-made covalab 21	1/500
	CHMP2B K6me1	Home-made ProteoGenix 662S	1/1000
	SMYD2	sc-393827	1/200
	Nup153	906201 Biolegend	1/1000
	alpha tubulin	T5168 Sigma	1/2000
	CAp24 HIV-1	National Institute for Biological Standards and Control Centralized Facility for AIDS Reagents (NIBCS); ARP366	1/1000
	GAPDH	Santa Cruz; sc-47724	1/1000
	anti-GFP-HRP	GeneTex; GTX26663	1/1000
	Anti-mouse-HRP	115-035-003 Jacksonimmuno	1/10000
	Anti-rabbit-HRP	111-035-003 Jacksonimmuno	1/10000
**Antibodies used for IF**	alpha tubulin	T5168 Sigma	1/2000
	CHMP2B	ProteinTech 12527	1/500
	CHMP2B me1	Home-made ProteoGenix 663S	1/30
	VPS4A	Sigma-Aldrich (SAB4200025)	1/200
	Anti-rabbit-488	invitrogen A11008	1/500
	Anti-mouse-594	R37121 Thermofisher	1 drop for 1ml
	Anti-rabbit-568	invitrogen A11011	1/500
	Anti-mouse-647	ProteoGenix IR003 E650	1/500
**Antibodies used for IP**	gfp	ab290 abcam	
	CHMP2B	ab226298	

Supplementary Table S5

Antibodies used in this study

Peptide Sequence and modifications (methylation, acetylation, and phosphorylation addition)	MS1 Mass [m/z]	Charge state	NCE
KKKTVDDVIKEQNREL	486.5285	4	30
	648.3689	3	
	972.04969	2	
KKKTVDDVIKEQNREL + me	490.03242	4	30
	653.04078	3	
	979.05751	2	
KKKTVDDVIKEQNREL + me + P	510.024	4	27
	679.69623	3	
KKKTVDDVIKEQNREL + 2 x me	493.53633	4	30
	657.71267	3	
	986.06534	2	
KKKTVDDVIKEQNREL + 2 x me + P	513.52791	4	27
	684.36811	3	
KKKTVDDVIKEQNREL + 3 x me	497.04024	4	30
	662.38455	3	
	993.07316	2	
KKKTVDDVIKEQNREL + 3 x me + P	517.03183	4	27
	689.03999	3	
KKKTVDDVIKEQNREL + ac	497.03115	4	27
	662.37242	3	
KKKTVDDVIKEQNREL + ac + P	517.02273	4	27
	689.02786	3	
KKKTVDDVIKEQNREL + P	506.52009	4	27
	675.02434	3	

Supplementary Table S6

PRM peptides used to characterize CHMP2B

Peptide Sequence and modifications	MS1 Mass [m/z]	Charge state	Extracted fragment ions	NCE
ADHYQQNTPIGDGPVLLPDNHY	8,220,558	3	y12, y10, y9, y7, y6, y5, y3, y2, b5,b6,b7, b8, b10, b13++	27
EGDTLVNRIEL	6,298,355	2	y10, y9, y8, y7, y6, y5, y4, y2, y1, b2, b3, b4, b5, b8, b9	27
KacKKTVDDVIKEQNREL	6,623,724	3	y8, y7, y6, y5, y4, y14++, y10++, y9++, y8++, y7++, b8, b9, b8++, b9++, b10++	30
KGIDFKEDGNIL	674,859	2	y11, y9, y8, y2, y1, b1, b2, b3, b4, b5, b6, b8, b10, b10++, b11++	27
KIRHNIEDGSVQLADHY	6,656,729	3	y11, y10, y9, y6, y5, y4, y3, y2, b6++, b7++, b8++, b10++, b11++, b12++, b15++	27
KKKTVDDVIKEQNREL	4,865,285	4	y8, y7, y6, y5, y4, y3, y14++, y13++, y11++, y10++, y9++, y8++, y7++, b2, b8	30
	6,483,689	3	y11, y8, y7, y6, y5, y4, y15++, y14++, y13++, y12++, y7++, b2, b8, b9, b8++	30
KmeKKTVDDVIKEQNREL	4,900,324	4	y8, y7, y6, y5, y4, y11++, y10++, y8++, y7++, b2, b3, b8, b7++, b8++, b9++	30
	6,530,407	3	y8, y7, y6, y5, y4, y14++, y8++, y7++, b2, b7, b8, b9, b7++, b8++, b9++	30
	9,790,575	2	y14, y13, y12, y11, y10, y9, y8, y7, y6, y14++, b2, b3, b6, b7, b15++	30
KdimeKKTVDDVIKEQNREL	6,577,126	3	y8, y7, y6, y5, y4, y10++, y9++, y7++, b6, b7, b8, b9, b7++, b8++, b9++	30
QQNTPIGDGPVLLPDNHY	6,599,937	3	y8, y7, y6, y5, y4, y3, y2, y5++, b4, b6, b8, b9, b11, b9++, b10++	27
	9,894,869	2	y16, y14, y12, y10, y9, y7, y6, y5, y4, y3, y2, y14++, b2, b3, b4	27
QQNTPIGDGPVLLPDNHYL	6,976,884	3	y8, y7, y6, y5, y4, y3, y6++, b4, b6, b8, b9, b11, b12, b9++, b10++	27
	10,460,289	2	y16, y15, y13, y11, y10, y8, y7, y6, y4, y3, y15++, b2, b3, b4, b8	27
RSRAQASNSAVDGTAGPSTSLY	7,326,928	3	y2, y1, y19++, y4++, b10++, b11++, b12++, b13++, b14++, b15++, b16++, b18++, b19++, b20++, b21++	27
	10,985,356	2	y11, y10, y8, y7, y6, b11, b12, b11++, b12++, b15++, b16++, b18++, b19++, b20++, b21++	27
SVSGEGEGDATYGKL	7,353,414	2	y13, y12, y11, y10, y9, y8, y7, y6, y5, y4, y3, y2, y3++, b2, b3	27
SVSGEGEGDATYGKLTL	8,424,072	2	y15, y14, y12, y11, y10, y8, y7, y6, y5, y4, y2, b2, b3, b5, b15	27
TGVVPILVELDGDVNGHKF	6,703,616	3	y13, y12, y11, y10, y9, y8, y6, y5, y2, y16++, y15++, y13++, b2, b3, b4	27
	10,050,387	2	y15, y13, y12, y11, y10, y9, y8, y6, y5, y15++, y13++, b2, b3, b4, b7	27
VNRIELKGIDFKEDGNIL	6,917,161	3	y2, b5, b6, b7, b10, b8++, b9++, b10++, b11++, b12++, b13++, b14++, b15++, b16++, b17++	27

Supplementary Table S7
PRM peptides used to quantify CHMP2B K6 modification

Peptide sequence and modifications	Targeted ions		Extracted fragment ions
	Mass [m/z]	Charge State	
KKKTVDDVIKEQNREL	486.5285	4	y3, y8++, y7++
	648.3689	3	y8++, y7++, b7++, b8++
KKKTVDDVIKEQNREL + Me	490.0324	4	y7++, b5++, b7++, b8++
	653.0407	3	y7++, b2, b7++, b8++, b9++
KKKTVDDVIKEQNREL +2 Me	493.5363	4	b3++, b5++, b7++, b 8++
	657.7126	3	y7++, b6++, b7++, b8++
KKKTVDDVIKEQNREL +3 Me	497.0402	4	y7++, b3++, b4++, b8++
	662.3845	3	y7++, b6++, b7++, b8++, b9++

Supplementary Table S8

PRM peptides used to estimate methylation rate of CHMP2B

Amount (pmol)			
KKKTVDDVIKEQNREL	KmeKKTVDDVIKEQNREL	KDimeKKTVDDVIKEQNREL	KTrimeKKTVDDVIKEQNREL
50	50	1,5	3
100	100	3	5
150	150	5	10
200	200	10	20
300	300	20	40
600	600	40	

Supplementary Table S9

CHMP2B peptides and amounts used to build the calibration curves
