# Supplementary material for: Methylation of ESCRT-III components regulates the timing of cytokinetic abscission
Source: Nat Commun. 2024 May 13;15:4023. doi: 10.1038/s41467-024-47717-3 (PMC11091153; doi:10.1038/s41467-024-47717-3)
Supplement: Supplementary file 3 — Description of additional supplementary files [file 41467_2024_47717_MOESM3_ESM.pdf]

Description of Additional Supplementary Files

Supplementary Movie 1 Live cell imaging of HeLa cell line transiently transfected with GFP_SMYD2, and treated with siR-tubulin (magenta). Left panel = GFP_SMYD2 alone, right panel = GFP_SMYD2 merge with Tubulin. Scale bar = 5tm, time is indicated in minutes.

Supplementary Movie 2 Phase contrast time-lapse videomicroscopy frames of HeLa cell line stably expressing GFP treated with control siRNA (siLuciferase). Empty arrow indicates the abscission. Scale bar = 10tm, time is indicated in minutes.

Supplementary Movie 3 Phase contrast time-lapse videomicroscopy frames of HeLa cell line stably expressing GFP treated with CHMP2B siRNA. Empty arrow indicates the abscission. Scale bar = 10tm, time is indicated in minutes.

Supplementary Movie 4 HeLa cell lines stably expressing siRNA resistant versions of CHMP2B_GFP treated CHMP2B siRNA. Empty arrow indicates the abscission. Scale bar = 10tm, time is indicated in minutes.

Supplementary Movie 5 HeLa cell lines stably expressing siRNA resistant versions of CHMP2B K6A_GFP treated CHMP2B siRNA. Empty arrow indicates the abscission. Scale bar = 10tm, time is indicated in minutes.

Supplementary Movie 6 Live cell imaging of HeLa cell line stably expressing CHMP2B_GFP treated with siR-tubulin (magenta). The empty arrows indicate the arrival of CHMP2B_GFP at the midbody, the transition from ring structures to midbody arms, and the cutting of microtubules used for calculating the6 duration of each abscission stage. Scale bar = 5tm, time is indicated in minutes.

Supplementary Movie 7 Live cell imaging of HeLa cell line stably expressing CHMP2B K6A_GFP treated with siR-tubulin (magenta). The empty arrows indicate the arrival of CHMP2B_GFP at the midbody, the transition from ring structures to midbody arms, and the cutting of microtubules used for calculating the duration of each abscission stage. Scale bar = 5tm, time is indicated in minutes.

Supplementary Movie 8 Live cell imaging of HeLa cell line stably expressing CHMP2B_GFP treated with DMSO (control). Tubulin was stained with siR-tub (magenta). The empty arrows indicate the arrival of CHMP2B_GFP at the midbody, the transition from ring structures to midbody arms, and the cutting of microtubules used for calculating the duration of each abscission stage. Scale bar = 5tm, time is indicated in minutes.

Supplementary Movie 9 Live cell imaging of HeLa cell line stably expressing CHMP2B_GFP treated with SMYD2 inhibitor (BAY-598 10tM). Tubulin was stained with siR-tub (magenta). The empty arrows indicate the arrival of CHMP2B_GFP at the midbody, the transition from ring structures to midbody arms, and the cutting of microtubules used for calculating the duration of each abscission stage. Scale bar =5tm, time is indicated in minutes.

Supplementary Movie10 Live cell imaging of HeLa cell line stably expressing CHMP2B_GFP treated with treated with control siRNA. Tubulin was stained with siR-tub (magenta). The empty arrows indicate the arrival of CHMP2B_GFP at the midbody, the transition from ring structures to midbody arms, and the cutting of microtubules used for calculating the duration of each abscission stage. Scale bar = 5tm, time is indicated in minutes.

Supplementary Movie 11 Live cell imaging of HeLa cell line stably expressing CHMP2B_GFP treated with treated with SMYD2 siRNA. Tubulin was stained with siR-tub (magenta). The empty arrows indicate the arrival of CHMP2B_GFP at the midbody, the transition from ring structures to midbody arms, and the cutting of microtubules used for calculating the duration of each abscission stage. Scale bar = 5tm, time is indicated in minutes.

Supplementary Movie 12 Live cell imaging of HeLa cell line stably expressing CHMP2B K6A_GFP treated with control siRNA. Tubulin was stained with siR-tub (magenta). The empty arrows indicate the arrival of CHMP2B_GFP at the midbody, the transition from ring structures to midbody arms, and the cutting of microtubules used for calculating the duration of each abscission stage. Scale bar = 5tm, time is indicated in minutes.

Supplementary Movie 13 Live cell imaging of HeLa cell line stably expressing CHMP2B K6A_GFP treated with SMYD2 siRNAs. Tubulin was stained with siR-tub (magenta). The empty arrows indicate the arrival of CHMP2B_GFP at the midbody, the transition from ring structures to midbody arms, and the cutting of microtubules used for calculating the duration of each abscission stage. Scale bar = 5tm, time is indicated in minutes.

Supplementary Movie 14 Live cell imaging of HeLa cell line stably expressing CHMP2B_GFP with Cherry. Tubulin was stained with siR-tub (magenta). The empty arrows indicate the arrival of CHMP2B_GFP at the midbody, the transition from ring structures to midbody arms, and the cutting of microtubules used for calculating the duration of each abscission stage. Scale bar = 5tm, time is indicated in minutes.

Supplementary Movie 15 Live cell imaging of HeLa cell line stably expressing CHMP2B_GFP with Cherry_SMYD2. Tubulin was stained with siR-tub (magenta). The empty arrows indicate the arrival of CHMP2B_GFP at the midbody, the transition from ring structures to midbody arms, and the cutting of microtubules used for calculating the duration of each abscission stage. Scale bar = 5tm, time is indicated in minutes.

Supplementary Movie 16 Phase contrast time-lapse videomicroscopy frames of HeLa cell lines stably expressing GFP treated with Nup153 siRNA. Empty arrow indicates the abscission. Scale bar = 10tm, time is indicated in minutes.

Supplementary Movie 17 Phase contrast time-lapse videomicroscopy frames of HeLa cell lines stably expressing SMYD2_GFP treated with Nup153 siRNA. Empty arrow indicates the abscission. Scale bar = 10tm, time is indicated in minutes.

Supplementary Movie 18 Fluorescent videomicroscopy frames of chromatin bridge in HeLa cell lines stably expressing GFP with lap2_ I3_RFP merge with phase contrast videomicroscopy frames. The three empty arrows show chromatin bridges. Empty arrow on merged videos indicates the time point prior the abscission. Left panel = lap2_ I3_RFP alone, right panel = lap2_ I3_RFP merge with phase contrast acquisitions.

Supplementary Movie 19 Fluorescent videomicroscopy frames of chromatin bridge in HeLa cell lines stably expressing SMYD2_GFP with lap2_ β_RFP merge with phase contrast videomicroscopy frames. The three empty arrows show chromatin bridges. Empty arrow on merged videos indicates the time point prior the abscission. Left panel = lap2_ f3_RFP alone, right panel = lap2_ f3_RFP merge with phase contrast acquisitions.

Supplementary Movie 20 Phase contrast time-lapse videomicroscopy frames of HeLa cell lines stably expressing GFP treated with Rab35 siRNA. Empty arrow indicates the abscission. Scale bar = 10µm, time is indicated in minutes.

Supplementary Movie 21 Phase contrast time-lapse videomicroscopy frames of HeLa cell lines stably expressing SMYD2_GFP treated with Rab35 siRNA. Empty arrow indicates the abscission. Scale bar = 10µm, time is indicated in minutes.
