# Supplementary material for: Methylation of ESCRT-III components regulates the timing of cytokinetic abscission
Source: Nat Commun. 2024 May 13;15:4023. doi: 10.1038/s41467-024-47717-3 (PMC11091153; doi:10.1038/s41467-024-47717-3)
Supplement: Supplementary file 25 — Reporting summary [file 41467_2024_47717_MOESM25_ESM.pdf]

nature portfolio

Corresponding author(s): Souhila Medjkane

Last updated by author(s): Feb, 8th 2024

# Reporting Summary

Nature Portfolio wishes to improve the reproducibility of the work that we publish. This form provides structure for consistency and transparency in reporting. For further information on Nature Portfolio policies, see our Editorial Policies and the Editorial Policy Checklist.

## Statistics

For all statistical analyses, confirm that the following items are present in the figure legend, table legend, main text, or Methods section.

| n/a | Confirmed | |
|---|---|---|
| ☐ | ☒ | The exact sample size (*n*) for each experimental group/condition, given as a discrete number and unit of measurement |
| ☐ | ☒ | A statement on whether measurements were taken from distinct samples or whether the same sample was measured repeatedly |
| ☐ | ☒ | The statistical test(s) used AND whether they are one- or two-sided<br>*Only common tests should be described solely by name; describe more complex techniques in the Methods section.* |
| ☒ | ☐ | A description of all covariates tested |
| ☐ | ☒ | A description of any assumptions or corrections, such as tests of normality and adjustment for multiple comparisons |
| ☐ | ☒ | A full description of the statistical parameters including central tendency (e.g. means) or other basic estimates (e.g. regression coefficient) AND variation (e.g. standard deviation) or associated estimates of uncertainty (e.g. confidence intervals) |
| ☐ | ☒ | For null hypothesis testing, the test statistic (e.g. *F*, *t*, *r*) with confidence intervals, effect sizes, degrees of freedom and *P* value noted<br>*Give P values as exact values whenever suitable.* |
| ☒ | ☐ | For Bayesian analysis, information on the choice of priors and Markov chain Monte Carlo settings |
| ☒ | ☐ | For hierarchical and complex designs, identification of the appropriate level for tests and full reporting of outcomes |
| ☒ | ☐ | Estimates of effect sizes (e.g. Cohen's *d*, Pearson's *r*), indicating how they were calculated |

*Our web collection on statistics for biologists contains articles on many of the points above.*

## Software and code

Policy information about availability of computer code

| | |
|---|---|
| Data collection | Microscopy data collection with MetaMorph software (version 7.10.3.279). Image Studio Lite (version 5.2) was used for western blot acquisition. |
| Data analysis | Inkscape (version 0.92.3) was used for making Figures. GraphPad Prism (version 9) was used for statistic. Image Studio Lite (version 5.2) was used for western blot quantification. Image analysis was performed using the Fiji software ( version 2.9.0/1.53t). |

For manuscripts utilizing custom algorithms or software that are central to the research but not yet described in published literature, software must be made available to editors and reviewers. We strongly encourage code deposition in a community repository (e.g. GitHub). See the Nature Portfolio guidelines for submitting code & software for further information.

## Data

Policy information about availability of data

All manuscripts must include a data availability statement. This statement should provide the following information, where applicable:

- Accession codes, unique identifiers, or web links for publicly available datasets
- A description of any restrictions on data availability
- For clinical datasets or third party data, please ensure that the statement adheres to our policy

All data are available in the main text or the supplementary materials.65
The mass spectrometry proteomics data have been deposited to the ProteomeXchange Consortium (http://proteomecentral.proteomexchange.org) via the PRIDE partner repository62 with the dataset identifier PXD041760 (https://ftp.pride.ebi.ac.uk/pride/data/archive/2024/03/).

## Research involving human participants, their data, or biological material

Policy information about studies with human participants or human data. See also policy information about sex, gender (identity/presentation), and sexual orientation and race, ethnicity and racism.

| | |
|---|---|
| Reporting on sex and gender | NA |
| Reporting on race, ethnicity, or other socially relevant groupings | NA |
| Population characteristics | NA |
| Recruitment | NA |
| Ethics oversight | NA |

Note that full information on the approval of the study protocol must also be provided in the manuscript.

# Field-specific reporting

Please select the one below that is the best fit for your research. If you are not sure, read the appropriate sections before making your selection.

- [x] Life sciences
- [ ] Behavioural & social sciences
- [ ] Ecological, evolutionary & environmental sciences

For a reference copy of the document with all sections, see nature.com/documents/nr-reporting-summary-flat.pdf

# Life sciences study design

All studies must disclose on these points even when the disclosure is negative.

| | |
|---|---|
| Sample size | No statistical method was used to predetermine sample size. |
| Data exclusions | No data exclusion. |
| Replication | Reproducibility was confirmed.The number of experiments is described in each Figure legend (N>=3 independent experiments) |
| Randomization | We assessed cells from multiple fields for each experiment, the fields were chosen randomly. The number of cells in each experiment is indicated in the relevant Figure Legends. |
| Blinding | The investigators were not blinded to the sample ID during experiments. |

# Reporting for specific materials, systems and methods

We require information from authors about some types of materials, experimental systems and methods used in many studies. Here, indicate whether each material, system or method listed is relevant to your study. If you are not sure if a list item applies to your research, read the appropriate section before selecting a response.

## Materials & experimental systems

| n/a | Involved in the study |
|---|---|
| ☐ | ☒ Antibodies |
| ☐ | ☒ Eukaryotic cell lines |
| ☒ | ☐ Palaeontology and archaeology |
| ☒ | ☐ Animals and other organisms |
| ☒ | ☐ Clinical data |
| ☒ | ☐ Dual use research of concern |
| ☒ | ☐ Plants |

## Methods

| n/a | Involved in the study |
|---|---|
| ☒ | ☐ ChIP-seq |
| ☒ | ☐ Flow cytometry |
| ☒ | ☐ MRI-based neuroimaging |

# Antibodies

| | |
|---|---|
| Antibodies used | The following antibodies were used in this study (Table S5): GFP (ab290 abcam, WB/IP), FLAG (Sigma A 8592, WB), CHMP2B (ab226298 abcam, WB/IP), CHMP2B (ProteinTech 12527, IF), CHMP2B me1 (Home made covalab 21, WB ; Home made ProteoGenix 662S, WBWB ; Home made ProteoGenix 663S, IF), VPS4A (Sigma SAB4200025, IF), alpha tubulin (T5168 Sigma, IF, WB), SMYD2 (Santa Cruz sc-393827, WB), CAp24 HIV-1 (National Institute for Biological Standards and Control Centralized Facility for AIDS Reagents (NIBCS); ARP366, WB), Nup153 (906201 Biolegend, WB). |
| Validation | CHMP2B ProteinTech 12527, home made CHMP2B-K6me1 proteoGenix 663S antibodies were validated by siRNA experiments, SMYD2 inhibitor or SMYD2 gain of function in this study using IF (Figures 2A-E, S2A-E, S5B). CHMP2B ab226298 abcam, home made CHMP2B-K6me1 covalab 21 and home made CHMP2B-K6me1 ProteoGenix 662S, Nup153 and SMYD2 antibodies were validated by siRNA experiments/expression of CHMP2B_K6A mutant/SMYD2 inhibitor in this study using WB (Figures 3A, 4A, 5A, S2A, S5A). |

# Eukaryotic cell lines

Policy information about cell lines and Sex and Gender in Research

| | |
|---|---|
| Cell line source(s) | HeLa cells were from American Type Culture Collection (ATCC) Clone CCL2, other cell lines were derived from this parental cell line. U2OS cells were from American Type Culture Collection (ATCC). |
| Authentication | Cell lines have not been authenticated. |
| Mycoplasma contamination | Cells were tested negative for mycoplasma contamination. |
| Commonly misidentified lines (See ICLAC register) | No commonly mis-identified cell lines were used in this study. |

# Plants

| | |
|---|---|
| Seed stocks | *Report on the source of all seed stocks or other plant material used. If applicable, state the seed stock centre and catalogue number. If plant specimens were collected from the field, describe the collection location, date and sampling procedures.* |
| Novel plant genotypes | *Describe the methods by which all novel plant genotypes were produced. This includes those generated by transgenic approaches, gene editing, chemical/radiation-based mutagenesis and hybridization. For transgenic lines, describe the transformation method, the number of independent lines analyzed and the generation upon which experiments were performed. For gene-edited lines, describe the editor used, the endogenous sequence targeted for editing, the targeting guide RNA sequence (if applicable) and how the editor was applied.* |
| Authentication | *Describe any authentication procedures for each seed stock used or novel genotype generated. Describe any experiments used to assess the effect of a mutation and, where applicable, how potential secondary effects (e.g. second site T-DNA insertions, mosiacism, off-target gene editing) were examined.* |
